# Noninvasive Human-Computer Interface Methods and Applications for Robotic Control: Past, Current, and Future

**DOI:** 10.1155/2022/1635672

**Published:** 2022-06-08

**Authors:** Xiaomei Hu, Yajuan Liu, Hao Lan Zhang, Wei Wang, Yijie Li, Chao Meng, Zhengke Fu

**Affiliations:** Center for SCDM, School of Media and Law, NingboTech University, Ningbo 315100, China

## Abstract

The purpose of this study is to explore the noninvasive human-computer interaction methods that have been widely used in various fields, especially in the field of robot control. To have a deep understanding of the development of the methods, this paper employs “Mapping Knowledge Domains” (MKDs) to find research hotspots in the area to show the future potential development. Through the literature review, this paper found that there was a paradigm shift in the research of noninvasive BCI technologies for robotic control, which has occurred from early 2010 since the rapid development of machine learning, deep learning, and sensory technologies. This study further provides a trend analysis that the combination of data-driven methods with optimized algorithms and human-sensory-driven methods will be the key areas for the future noninvasive method development in robotic control. Based on the above findings, the paper provides a potential developing way of noninvasive HCI methods for related areas including health care, robotic system, and media.

## 1. Introduction

This paper presents a comprehensive review of noninvasive HCI methods and application for robotic control. Robotic systems and their applications have been applied extensively in various areas including medical clinics, physiological exercises, and manufacturing. Noninvasive Brain-Computer Interface (BCI) research, e.g. EEG-related robotic control, has become one of the most important topics in HCI areas since it can minimize potential risks and provide high time resolution. Many cutting-edge technologies have been utilized in analyzing EEG data, such as common spatial patterns (CPSs) [1], time series analysis, graph convolutional networks (GCNs), and other machine learning methods. In order to draw a comprehensive picture on the development of the non-invasive/wearable methods for robotic system control, we conducted a systematic survey on noninvasive HCI technologies and their application.

This paper has four parts.  Section 1 reviews the existing research bases and research hotspots on noninvasive robotic control by using the knowledge graph tool.  Section 2 presents noninvasive HCI methods for robotic control from 1960s to 2010, mainly focusing on brain-related robotic/machine control, including EEG-related robotic/machine control and other HCI control.  Section 3 provides a concrete survey on the recent development of noninvasive HCI technologies and applications from 2010 to 2021, mainly focusing on artificial intelligence/machine learning methods for robotic control based on EEG/MEG/MRI/fMRI, theoretical development on EEG/MEG/MRI for robotic in recent years. In Section 4, we conclude our research findings based on the extensive survey provided in Section 2 and Section 3, and provide a trend analysis on future noninvasive HCI methods for robotic control and their applications.

## 2. Overview

**Figure 1:**
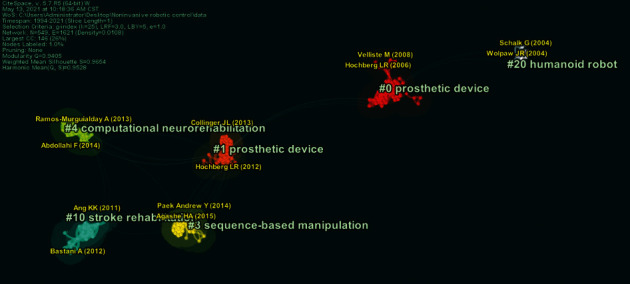
Fundamental analysis of research based on literature.

In this paper, data-intensive scientific discoveries and “Mapping Knowledge Domains” (MKD) were used for research, and the SSCI database in the core papers of the Web of Science (WOS) database was collected as the source channel of samples. Using noninvasive robotic control as the key words, a total of 137 entries containing all the record fields and references were retrieved from the whole database in the time range of subject fields (from January 1994 to May 2021, with the time slice of 1). The threshold value of each time slice is g-index (*k* = 25). Finally, the structure, rule, and distribution of scientific knowledge are presented through visualization. The network is divided into 6 co-citation clusters. These clusters are labeled by index terms from their own citers (see Figure 1).

### 2.1. Research Hot Spots

**Figure 2:**
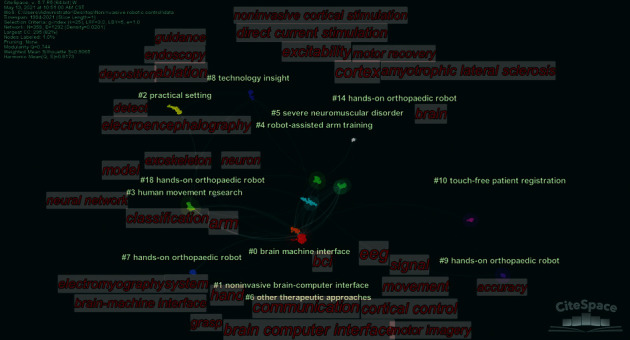
Research hotspots and Frontier analysis based on literature.

In this paper, we conducted a comprehensive survey on the research hot spots based on Citespace software. The network is divided into 13 co-citation clusters which are labeled by index terms from their own citers also (see Figure 2).

### 2.2. Emerging Noninvasive BCI-Robotic Control Technologies

In recent years, the noninvasive BCI-robotic-control research direction has been shifted from signal processing and pattern recognition to machine learning and neuron computing. Their applications have been focused on wearable devices and health-related HCI devices, as shown in Figures 1 and 2.

Chung et al. (2011) demonstrated a new adaptive and hierarchical approach to BCI which made the control of complex robotic devices faster and more accurate. After comparing with 2D, LaFleur et al. (2013) indicated that noninvasive EEG-based BCI systems could be used well in 3D physical space for complex control. Ibanez et al. (2013) proposed an adaptive and asynchronous system based on EEG to detect online the purpose of moving in tremor patients. Comparison were made between healthy people and patients, adaptive design, and fixed design, and the adaptive design was found to have a higher number of movement detections. Dong et al. (2018) presented a way to decode cortical potentials of lower-limb movements based on continuous classification and asynchronous detection. Terada et al. (2015) developed a wearable EEG-based brain robot interface (WE-BRI). The interface uses steady-state visual evoked potential (SSVEP) to detect people's intention of movements.

## 3. A Survey on Robotic Control Applications Using Noninvasive Methods (1990–2010)

### 3.1. Robotic Control in the 1990s

The robotic control research has been a crucial area in the twentieth century, and it remains an important discipline in this century. In the early research on brain-related robotic control systems, a big problem which hindered the development of BCI-robotic control was the noise of the brain signals as well as the limitations of algorithmic and mechanical technologies.

In order to solve the problems in the traditional robotic control system development, some study develops the robotic control system in a new way, including Petri nets, neural network, and algorism platform. Based on Petri nets which benefit from the Petri nets technique [2], Caloini et al. proposed an approach to design robotic controller. Yu et al. proposed a platform for robotic control algorithm [3]. They recommend that the adaptive computing system, with its good performance and high dependability, is a suitable platform to implement algorism for robotic control. With this improvement, robotic control had been applied in different areas. For example, in the medical environment, it had been used in knee joint replacement surgery [4] and tissue surgery [5]. Besides, they had been used in deep ocean detection [6] and telescope development [7]. Barreto et al. proposed a robot control system using competitive and temporal Hebbian (CTH) network, which applies temporal self-organizing neural network [8]. By utilizing two sets of individual states, the individual states of the trajectory, and the temporal order of trajectory states, this network can utilize two sets of synaptic weights. The neural network is also used in vision system development.

### 3.2. Early Noninvasive BCI Methods for Robotics Control

**Figure 3:**
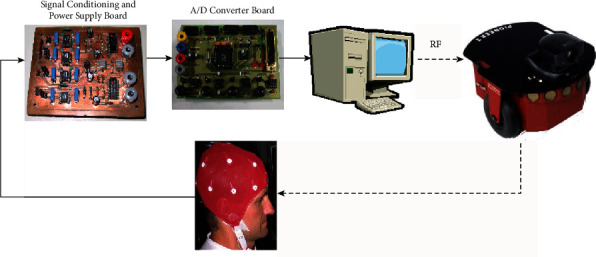
A proposed HMI [14].

Noninvasive BCI methods for robotic control have become a hot topic since 2004, particularly the EEG-based robotics. Various experimental results show that noninvasive EEG-based BCI could control movements of a mobile robot or a robotic arm by brain signals collected from scales through EEG [9]. [10]. In 2007, Kayagil et al. explored a binary approach using the binary cursor control paradigm to achieve more complex controls by simply answering yes or no [11]. Wu and others designed an Ethernet robot to implement actions for disabled patients [12]. RFID (radio frequency identification) technologies were employed to help disables control medical robotics and neurorobotic prosthetics [13]. Functions of HMIs (human-machine interfaces) using EEG and EMG, respectively, were compared in an experiment too and the EEG-based HMI was proved to be an evolution of the EMG-based HMI (see Figure 3). EEG-based MI-BCI which can get robotic feedback from neuro rehabilitation was also proved to have advantages over robotic rehabilitation in restoring the motor function of upper extremities of hemiparetic stroke patients [15].

Among these methods, LDA (linear discriminant analysis) is one of the most popular mechanical systems for classification. In Fisher's study, he presented a process: first, we calculate a different linear function of the attributes for each class to be identified, and then the class function which yielded the highest score represented the predicted class. For the case that the scalar *α* is given by the quadratic form [16]:

(1) $$\begin{array}{c} \alpha =x^{T}Ax, \end{array}$$

where *x* is *n* ×  1, A is *n*  ×  *n*, and A does not depend on *x*, then

(2) $$\begin{array}{c} \;\frac{\partial \alpha }{\partial x}=x^{T}\left(A+A^{T}\right). \end{array}$$

## 4. Recent Development of Noninvasive Methods for Robotic Control (2010–2021)

### 4.1. The Noninvasive Robotic Control

Dan et al. pointed out that the noninvasive robotic control has its specific background for usage. Taking the brain-computer interfaces as an example, it enables device control through brain signals, which dramatically improves the life quality of disabled individuals [17]. For example, it can be used for stroke patients. In some study, BCI and robotic arm are combined to assist the after-stroke rehabilitation [18]. It also can be used in certain injury circumstances like Spinal Cord Injury [19]. There are many different ways to make nonintrusive control of the robotic, such as using the gloves. A study introduces how a wireless data glove can be used to control a robot [20]. The mechanism has two steps: first, the unit can translate the hand postures into data, and then the data will be sent to a unit to control it. Melidis et al. proposed the human-centric control methods [21]. The so-called human-centric control methods mean building an interface, which will translate the human behavior into robot action. Such interface makes remote control possible. Also, the human-machine interface system can use other signals from the human body; for example, tongue-movement ear pressure (TMEP)-based signal can send real-time signal [22]. A certain data mining model to optimize data sampling which includes the segmented EEG graph and the EEG-based weighted network was proposed [23].

### 4.2. EEG-Based Robotics

During 2011–2021, researchers continued to explore application of EEG-based robotics in different areas especially in medical area. Their research mainly focused on the application of EEG-based robotics, technological improvement, and assessment of the technology.

#### 4.2.1. Application

Medical area. Many studies found that EEG-based robotics could be largely used in rehabilitation of patients who had difficulties in their actions such as paralyzed people and stroke patients [24–26]. Ang and Chua also pointed out that it was effective for chronic stroke patients who had upper-limb hemiparesis to use an EEG-based MI-BCI system [27].

Other areas. Researcher also discussed application of EEG-based robotics in some other areas. Based on EEG, Overmeyer and Podszus provided a new cognitive approach combining speech and gesture control for multimodal HMI to be used in automated guided vehicles (AGVs) in logistics [28].

#### 4.2.2. Technological Improvement

Further exploration and optimization of the related technologies were made.

Detailed technological analysis of EEG and BCI was widely discussed [29–32], including application of augmented reality, computer vision, and SSVEP-BCI.

Especially in recent several years, researchers focused more on optimization of technology. Ogino and Mitsukura developed an emotion analyzer which could be used in a robotic arm system [33]. Korovesis et al. presented a system using alpha brain waveforms to get a synchronous and endogenous EEG-BCI which could help control a mobile robot with the eye's blinking of the subject [34]. Zhang et al. proposed an optimized data sampling model which could be used to further identify the status of human brain [23].

### 4.3. Noninvasive Mind-Controlled Robotic Arms

A robotic arm is a flexible mechanical device which has a similar function to the human arm. Mind-controlled robotic arms can help people who have body disabilities accomplish daily tasks, such as drinking and eating. Noninvasive BCI systems capture signals from the head scalp and then translate the signals into motion commands. A robotic arm, a prosthetic limb, or an exoskeleton can perform tasks as commands to simulate the human arm's function or to rehabilitate the neurologically disabled patients.

**Figure 4:**
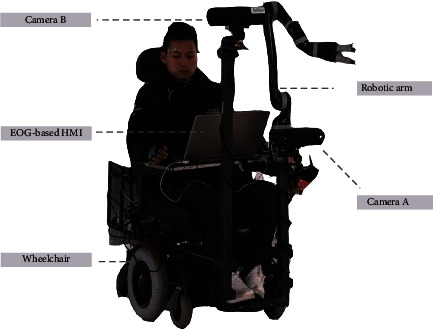
The combined system [39].

Some researchers developed hybrid BCI systems to improve the accuracy of these systems. For example, Pfurtscheller et al. combined ERD/ERS- and SSVEP-based BCIs [35]. Úbeda et al. combined a BCI with the RFID technology [36]. Gao et al. developed a robotic arm system that combines MI, EMG, and SSVEP to accomplish a writing task [37]. Xu et al. developed a MI-based BCI with the computer vision guidance [38]. And the robotic arm systems can be integrated with other systems to perform complex actions such as walking and grasping. For example, Huang et al. put a robotic arm to a wheelchair through which a patient can control his/her motions by an EOG-/EEG-based HMI (see Figure 4).

Intelligent robotic systems have been used in manipulating robotic arms. Zhang et al. developed a semiautonomous intelligent robotic system driven by intention. With the system, disabled patients can use the P300 system to send an intention command for one drinking task and the autonomous robot completes the rest [40]. Other EEG signals have also been applied to control a robotic arm. For example, Sharma K et al. employed blinks and teeth clenching to manipulate a robotic arm in 3D [41]. And Zeng et al. developed a hybrid BCI system, which combines an EEG signal-based BCI and an eye tracking system [42].

### 4.4. Noninvasive Brain Control

At present, research studies on noninvasive control brain are mainly focused on two aspects.

#### 4.4.1. Rehabilitation Training Was Conducted through Noninvasive Brain Control

Recently, the relevant rehabilitation training through noninvasive brain stimulation has been focused on the functional recovery and conditions of patients after stroke, migraine, etc., and most of the studies have supported that certain noninvasive stimulation can have a positive effect on some symptoms. After a systematic review and meta-analysis, Kang et al. confirmed that NIBS may be an effective way to restore functional balance and postural control of stroke patients [43]. Brabenec, et al. suggested that transcranial direct current stimulation (TDCS) could improve the recovery of poststroke [44]. They discussed whether the dual-TDCS of the primary motor cortex would improve the learning and retention skills of stroke patients. Enhancing the exciting level of the motor cortex through repeated transcranial magnetic stimulation (RTMS) appears to be a well-tolerated and effective strategy for motor recovery early after acute stroke. In contrast, early transcranial direct current stimulation (TDCS) after stroke did not promote motor recovery. However, in the chronic phase, both RTMS and TDCS have been shown to be beneficial when applied over several days in combination with training. Although noninvasive brain stimulation appears to support motor recovery, it is noted that to date, there is a lack of robust randomized controlled trials (RCTs) [45].

A randomized double-blinded Sham controlled study showed that noninvasive brain stimulation with M1 enhanced hand strength control ability [46]. The research studies also revealed that noninvasive brain stimulation has certain curative effect for patients with migraine. The frontal noninvasive brain stimulation can improve the negative symptoms of schizophrenia, etc., through RTMS and TDCS [47] [48]. An experiment on exercise rehabilitation in children with brain injury showed that noninvasive brain stimulation has some effect on the treatment of movement disorders in children with brain injury [49]. It was found that NIBS can safely stimulate children with brain injury, RTMS can improve upper limb function, TDCS can improve balance, and most gait variables continue to act for 1 month. The efficacy of spasms is uncertain.

Earlier, Rogers et al. explored the feasibility of applications of noninvasive brain-computer interfaces (BCIs) to restore voluntary motor control for stroke patients, pointing out that the majority of stroke patients have persistent deficits and that current interventions fail to restore their normal motor behavior. Noninvasive brain-computer interfaces (BCIs) have the potential to offer restorative benefits. They also found some other potential advantages when they combined BCI with functional electrical stimulation (FES). The feasibility of combining the two for motor learning of stroke patients has also been tested [50].

Conversely, there have been studies which found that there had been no significant evidence to prove that noninvasive brain stimulation could have positive effects on neuropathic pain and depression for individuals with SCI. While, researchers found that cranial electrical stimulation might be beneficial for the treatment of anxiety disorders. Therefore, these findings do not support the routine use of noninvasive brain stimulation for neuropathic pain in patients with spinal cord injury [51].

#### 4.4.2. Control of Objects or Robots through BCI

Another important application of noninvasive control brain is the control of objects or robots, but the starting point of some studies is still the consideration of patient rehabilitation.

Lafleur et al. report novel experiments of BCI in human subjects using noninvasive scalp electroencephalography (EEG) to control a robotic quadcopter in 3D physical space, using metrics applicable to asynchronous BCI to quantify the performance of the system [52]. This work demonstrates the potential of noninvasive EEG-based BCI systems to achieve complex control in 3D physical space and can also serve as a framework for the study of multidimensional noninvasive BCI control in physical environments, with telepresence robotics being used. Escolano C. et al. previously reported a brain-driven intelligent reality system (brain-driven remote telepresence system) based on EEG, which can provide users with a sense of telepresence in a remote environment and access to Internet through mobile robots. The system relies on a P300-based brain-computer interface and a mobile robot which has autonomous navigation and camera orientation functions [53].

The research of Chae et al. (2011) proposed a navigation system for humanoid robots, based on asynchronous noninvasive BCI. The behavior of the navigation system was similar to that of humans. The evaluation of the results verified the feasibility and robustness of the proposed system [54]. Brain-computer interface provides a new communication method for people who suffer from neurological disorders and cannot contract their muscles easily. Researchers found that by employing a BCI patients might control a neuroprosthetic robot directly through their brain and could achieve virtual interaction with the environment consequently. Therefore, A BCI supporting multidimensional control is highly needed for a multidimensional robot. Related studies also show that an interface through EEG can be used to control a partially autonomous humanoid robot and to make the robot perform complex tasks such as walking to a desired location or picking up the targeted object [55]. EEG-based brain-computer interfaces can be employed to help people make complex interactions with the environment. The robots applied not only are equipped with the navigation system as before but also can manipulate and transport objects [56].

In addition, the control of two-dimensional motion signals through noninvasive brain-computer interfaces [57], the emulation of computer mouse control a non-invasive BCI [58] and EEG powered mobile robots [9] were also explored earlier, with some researchers suggesting that BCIs could help people with complete paralysis communicate with others and control their motions. Both noninvasive and invasive methods can be used in BCIs to receive signals sent by the brain which convey the intentions of the user. Although noninvasive BCIs can be readily used in some simple applications, it is generally believed that only invasive BCIs with electrodes implanted in the brain of a patient can make multidimensional controls of a robotic arm or neural prosthesis.

There is an obvious division between the recent and early studies on noninvasive control brain by combing of the above literature studies. Since 2013, the application of noninvasive brain control mainly focuses on the field of rehabilitation, especially the recovery of limb function of patients after stroke, which has become the focus of attention. Before that, to the early 2000s, the application of noninvasive control brain was mainly concerned with the control application of objects or machines, and few literature studies paid attention to the field of rehabilitation. Current research literature shows that the application of noninvasive brain control will become an important direction in the field of rehabilitation in the future. Through the noninvasive brain control technology, the combination of object or robot control and rehabilitation training may become the focus of research to solve the obstacles in daily life of hemiplegia or the disabled.

### 4.5. Wearable Robotics

Wearable device is a portable device, that connect all kinds of sensors, identification, and cloud services, etc., into peoples glasses, watches, bracelets, clothing, footwear, such as daily wear, so as to realize the expansion of user perception and bring great changes to our life. In this paper, we will summarize the research trends of wearable robot from five aspects: neural interface, soft wearable robot, sensor and driver technology, robot exoskeleton technology, and design and development of wearable robot system (see Table 1).

## 5. Conclusions

In this survey, we reviewed the development and applications of noninvasive BCI technologies for robotic control since 1990s. The literature review indicates that noninvasive BCI technologies for robotic control have experienced a steady growth in 1990s; and much of the research work in the BCI-robotic control area was focusing on signal processing and algorithmic optimization. The typical methods used during this period include self-organizing neural network, common spatial patterns, wavelet transform (WT), discrete wavelet transform (DWT), and linear discriminant analysis (LDA). These methods were generally applied to wheelchair controlling, simulated robots controlling, and manufacturing robotic arms.

The paradigm shift has occurred from early 2010s since the rapid development of machine learning, deep learning, and sensory technologies. We summarize the related wearable HCI methods for robotic control into the following categories based on their applications: neural interface, soft wearable robots, sensor and actuator technology, and robot exoskeleton design. The emerging noninvasive BCI technologies for robotic control basically can be summarized into the following three categories: (i) algorithm-driven methods, such as small-world neural network (SWNN), MLP neural network, graph convolutional network (GCN). (ii) human-sensory-driven methods, such as steady state visual evoked potentials (SSVEPs), MEG-based methods, electromyography (EMG)-based methods, and so on. (iii) data-driven methods, which include reinforcement learning approach, nonlinear model predictive control, nonlinear digital time-delay dynamic systems, and so on.

Based on our survey, we discovered that the noninvasive HCI methods for robotic control is becoming the conventional and trendy solutions. Much research work still focuses on eliminating the noise in signal processing stage; however, the paradigm shift indicates that more and more researchers have adopted neural computing and machine learning technologies to improve robotic control efficiency. The following technologies will be the key areas for the future non-\invasive method development in robotic control:

1. The combination of data-driven methods with optimized algorithms: Internet-of-Things (IoT) applications will demand more wearable robotic facilities in our daily life. Therefore, the future noninvasive-based HCI for robotic control model will heavily rely on large data analytical methods and their optimization models.
2. Human-sensory-driven methods: in near future, sensors attached to human bodies will become a common phenomenon. Robotic control will become a companion technology with sensory technologies, in particular, human body sensory hardware, such as EEG, MEG, and eye sensors, in paired with the robotic control model [73].

## Figures and Tables

**Table 1:** A review of the development of wearable robot research.

Category	Methods	Applications	References
Neural interface	Wearable EMG intention detection system	Family rehabilitation	[59]
	BMI	Users with limb mobility impairments	[60]
	Integration of BCI and AR	Rehabilitation of Autism patients	[61]

Soft wearable robots	A new soft wearable robotic suit	Help the elderly with daily activities and walking	[62]
	Wearable robotic device	Upper limbs due to TBI	[63]
	Robotic glove driven by PAMs, acquires EMG signals from the forearm	Hand rehabilitation	[64]

Sensor and actuator technology	Manipulator control system	A wearable robotic arm	[65]
	Wearable robot glove based on optical FMG drive controller	Disabilities of the hand	[66]
	Extra robotic fingers	Assist a user in bimanual object manipulation	[67]

Robot exoskeleton	Motion assistive robotic-exoskeleton	The upper limb	[68]
	A wearable exoskeleton robot	Construction workers work safely	[69]
	A wearable motorized hand exoskeleton	Home-based movement therapy	[70]

Design and development of wearable robot system	A subject-independent classification method, based on support vector machines	The identification of locomotion-related activities	[71]
	Wearable assistive robotic knee device, assist-as-needed control strategy	Additional support and actuation for human walkers	[72]
	Interaction-based assist-as-needed impedance	Ankle robotic orthosis	[72]

## Data Availability

The data used to support the findings of this study are available from the corresponding author upon request. All the literature referred in this paper are obtained from the Web of Science (WOS) database.

## References

[1] Xu Y., Wei Q., Zhang H. (2019). Transfer learning based on regularized common spatial patterns using cosine similarities of spatial filters for motor-imagery BCI. *Journal of Circuits, Systems, and Computers*.

[2] Caloini A., Magnani G., Pezze M. (1998). A technique for designing robotic control systems based on Petri nets. *IEEE Transactions on Control Systems Technology*.

[3] Yu S. Y., Saxena N., McCluskey E. J. (2000). *ACS Implementation of A Robotic Control Algorithm with Fault Tolerant Capabilities*.

[4] Davies B. L., Rodriguez y Baena F. M, Barrett A. R (2007). Robotic control in knee joint replacement surgery. *Proceedings of the Institution of Mechanical Engineers - Part H: Journal of Engineering in Medicine*.

[5] Adagolodjo Y., Goffin L., Mathelin M. D., Courtecuisse H. Inverse real-time Finite Element simulation for robotic control of flexible needle insertion in deformable tissues.

[6] Rife J. H., Rock S. M. (2006). Design and validation of a robotic control law for observation of deep-ocean jellyfish. *IEEE Transactions on Robotics*.

[7] Fraser S. N., Steele I. A. (2002). Object oriented design of the liverpool telescope robotic control system. *Proceedings of the Society of Photo-optical Instrumentation Engineer(SPIT)*.

[8] Barreto G. A., Araujo A. F. R., Ducker C., Ritter H. (2002). A distributed robotic control system based on a temporal self-organizing neural network. *IEEE Transactions on Systems, Man and Cybernetics, Part C (Applications and Reviews)*.

[9] Millan J. D., Renkens F., Mourino J., Gerstner W. (2004). Noninvasive brain-actuated control of a mobile robot by human EEG. *IEEE Transactions on Biomedical Engineering*.

[10] Bell C. J., Shenoy P., Chalodhorn R., Rao R. P. N. (2008). Control of a humanoid robot by a noninvasive brain-computer interface in humans. *Journal of Neural Engineering*.

[11] Kayagil T., Bai O., Lin P., Furlani S., Vorbach S., Hallett M. Binary EEG control for two-dimensional cursor movement: an online approach.

[12] Wu L. W., Liao H. C., Hu J. S., Lo P. C. (2008). Brain-controlled robot agent: an EEG-based eRobot agent. *Industrial Robot: International Journal*.

[13] Eleni A. Control of medical robotics and neurorobotic prosthetics by non-invasive Brain-Robot Interfaces via EEG and RFID technology.

[14] Ferreira A., Celeste W. C., Cheein F. A., Bastos-Filho T. F, Sarcinelli-Filho M, Carelli R (2008). Human-machine interfaces based on EMG and EEG applied to robotic systems. *Journal of NeuroEngineering and Rehabilitation*.

[15] Ang K. K., Guan C. T., Chua K. S. G. Clinical study of neurorehabilitation in stroke using EEG-based motor imagery brain-computer.

[16] Fisher R. A. (1936). The use of multiple measurements in taxonomic problems. *Annals of Eugenics*.

[17] Dan S., Signorile R. An exploration of the utilization of electroencephalography and neural nets to control robots.

[18] Wang C., Phua K. S., Ang K. K. A feasibility study of non-invasive motor-imagery BCI-based robotic rehabilitation for Stroke patients.

[19] Athanasiou A., Arfaras G., Xygonakis L., Kartsidis P., Pandria N. Commercial BCI control and functional brain networks in spinal cord injury: a proof-of-concept.

[20] Rakib F. H., Howlader S., Rahman M., Bhuiyan A. Y. (2019). Application of BIM based interoperability-A case study. *Journal of Logistics, Informatics and Service Science*.

[21] Rahman A. (2019). Challenges in privately joint-ventured project: a case study. *Journal of Logistics, Informatics and Service Science*.

[22] Vanaga R., Sloka B. (2020). Financial and capital market commission financing: aspects and challenges. *Journal of Logistics, Informatics and Service Science*.

[23] Zhang H. L., Lee S., Li X., He J. (2020). EEG self-adjusting data analysis based on optimized sampling for robot control. *Electronics*.

[24] Onose G., Grozea C., Anghelescu A. (2012). On the feasibility of using motor imagery EEG-based brain-computerinterface in chronic tetraplegics for assistive robotic arm control: a clinical test and long-term post-trial follow-up. *Spinal Cord*.

[25] Ang K. K., Guan C., Phua K. S. Transcranial direct current stimulation and EEG-based motorimagery BCI for upper limb stroke rehabilitation.

[26] Dakak S., Wahbeh F. (2020). Designing fast transportation network in damascus: an approach using flow capturing location allocation model. *Journal of Logistics, Informatics and Service Science*.

[27] Ang K. K., Chua K. S. G., Phua K. S. (2015). A randomized controlled trial of EEG-based motor imagery brain-computer interface robotic rehabilitation for stroke. *Clinical EEG and Neuroscience*.

[28] Overmeyer L., Podszus F., Dohrmann L. (2016). Multimodal speech and gesture control of AGVs, including EEG-based measurements of cognitive workload. *CIRP Annals*.

[29] Contreras-Vidal J. L., Grossman R. G. NeuroRex: a clinical neural interface roadmap for EEG-based brain machine interfaces to a lower body robotic exoskeleton.

[30] He W., Zhao Y. (2016). A wireless BCI and BMI system for wearable robots. *IEEE Transactions on System Man Cybernetics-Systems*.

[31] Zhao X. G., Chu Y. Q., Han J., Zhang Z. (2016). SSVEP-based brain-computer interface controlled functional electrical stimulation system for upper extremity rehabilitation. *IEEE Transactions on System Man Cybernetics-Systems*.

[32] Chen X. G., Huang X. S., Wang Y., Gao X. (2020). Combination of augmented reality based brain- computer interface and computer vision for high-level control of a robotic arm. *IEEE Transactions on Neural Systems and Rehabilitation Engineering*.

[33] Ogino M., Mitsukura G., Koulouras Y., Alexandridis A. An EEG-based robot arm control to express human emotions.

[34] Korovesis N., Kandris D., Koulouras G., Alexandridis A. (2019). Robot motion control via an EEG-based brain-computer interface by using neural networks and alpha brainwaves. *Electronics*.

[35] Pfurtscheller G., Solis-Escalante T., Ortner R., Linortner P., MullerPutz G. R. (2010). Self-paced operation of an SSVEP-based orthosis with and without an imagery-based “brain switch:” a feasibility study towards a hybrid BCI. *IEEE Transactions on Neural Systems and Rehabilitation Engineering*.

[36] Úbeda A., Iáñez E., Azorín J. M. (2013). Shared control architecture based on RFID to control a robot arm using a spontaneous brain–machine interface. *Robotics and Autonomous Systems*.

[37] Gao Q., Dou L., Belkacem A. N., Chen C. (2017). Noninvasive electroencephalogram-based control of a robotic arm for writing task using hybrid BCI system. *BioMed Research International*.

[38] Mekvabidze R. (2020). From business modeling to business management: an exploratory study of the optimal decision making on the modern university level. *Journal of Logistics, Informatics and Service Science*.

[39] Huang Q., Chen Y., Zhang Z. (2019). An EOG-based wheelchair robotic arm system for assisting patients with severe spinal cord injuries. *Journal of Neural Engineering*.

[40] Zhang Z., Huang Y., Chen S. (2017). An intention-driven semi-autonomous intelligent robotic system for drinking. *Frontiers in Neurorobotics*.

[41] Sharma K., Jain N., Pal P. (2017). Telemanipulation of a robotic arm using EEG artifacts. *International Journal of Mechatron Electr Comput Technol*.

[42] Hong Z., Wang Y., Wu C., Song A., Wen P. (2017). Closed-loop hybrid gaze brain-machine interface based robotic arm control with augmented reality feedback. *Frontiers in Neurorobotics*.

[43] Kang N., Lee R. D., Lee J. H., Hwang M. H. (2020). Functional balance and postural control improvements in patients with stroke after noninvasive brain stimulation: a meta-analysis. *Archives of Physical Medicine and Rehabilitation*.

[44] Rektorova I., Brabenec L., Klobusiakova P. (2020). Noninvasive brain stimulation to treat hypokinetic dysarthria in PD: a sham stimulation-controlled trial. *Movement Disorders*.

[45] Kashiwagi F. T., El Dib R., Gomaa H. (2018). Noninvasive brain stimulations for unilateral spatial neglect after stroke: a systematic review and meta-analysis of randomized and nonrandomized controlled trials. *Neural Plasticity*.

[46] Jin Y., Lee J., Oh S., Gimenez M. C. F., Yoon B. C. (2019). Noninvasive brain stimulation over the M1 enhances bimanual force control ability: a randomized double-blind sham-controlled study. *Journal of Motor Behavior*.

[47] Shirahige L., Melo L., Nogueira F., Rocha S., Silva K. M. (2016). Efficacy of noninvasive brain stimulation on pain control in migraine patients: a systematic review and meta-analysis. *Headache*.

[48] Aleman A., Enriquez-Geppert S., Knegtering H., Lange J. J. D. D. (2018). Moderate effects of noninvasive brain stimulation of the frontal cortex for improving negative symptoms in schizophrenia: meta-analysis of controlled trials. *Neuroscience & Biobehavioral Reviews*.

[49] Elbanna S. T., Elshennawy S., Ayad M. N. (2019). Noninvasive brain stimulation for rehabilitation of pediatric motor disorders following brain injury: systematic review of randomized controlled trials. *Archives of Physical Medicine and Rehabilitation*.

[50] Daly J. J., Cheng R., Rogers J., Litinas K., Hrovat K., Dohring M. (2009). Feasibility of a new application of noninvasive brain computer interface (BCI): a case study of training for recovery of volitional motor control after stroke. *Journal of Neurologic Physical Therapy*.

[51] Yu B., Qiu H., Li J., Zhong C., Li J. (2020). Noninvasive brain stimulation does not improve neuropathic pain in individuals with spinal cord injury. *American Journal of Physical Medicine & Rehabilitation*.

[52] LaFleur K., Cassady K., Doud A., Shades K., Rogin E., He B. (2013). Quadcopter control in three-dimensional space using a noninvasive motor imagery-based brain-computer interface. *Journal of Neural Engineering*.

[53] Escolano C., Antelis J. M., Minguez J. (2012). A telepresence mobile robot controlled with a noninvasive brain-computer interface. *IEEE Transactions on Systems, Man, and Cybernetics - Part B: Cybernetics*.

[54] Chae Y., Jeong J., Jo S. Noninvasive Brain-Computer Interface-based control of humanoid navigation.

[55] Chen X., Bai O. Towards multi-dimensional robotic control via noninvasive brain-computer.

[56] Bell C. J., Shenoy P., Chalodhorn R., Rao R. P. N. (2008). Control of a humanoid robot by a noninvasive brain–computer interface in humans. *Journal of Neural Engineering*.

[57] Wolpaw J. R., McFarland D. J., Bizzi E. (2004). Control of a two-dimensional movement signal by anoninvasive brain-computer interface in humans. *Proceedings of the National Academy of Sciences - PNAS*.

[58] McFarland D. J., Krusienski D. J., Sarnacki W. A., Wolpaw J. R. (2008). Emulation of computer mouse control with a noninvasive brain–computer interface. *Journal of Neural Engineering*.

[59] Ryser F., Butzer T., Held J. P., Lambercy O., Gassert R. Fully embedded myoelectric control for a wearable robotic hand orthosis.

[60] Baldi T. L., Spagnoletti G., Dragusanu M., Prattichizzo D. Design of a wearable interface for lightweight robotic arm for people with mobility impairments.

[61] Arpaia P., Bravaccio C., Corrado G., Duraccio L., Moccaldi N., Rossi S. Robotic autism rehabilitation by wearable brain-computer interface and augmented reality.

[62] Jin S., Iwamoto N., Hashimoto K., Yamamoto M. (2017). Experimental evaluation of energy efficiency for a soft wearable robotic suit. *IEEE Transactions on Neural Systems and Rehabilitation Engineering*.

[63] Kadivar Z., Beck C. E., Rovekamp R. N., Malley M. K. O., Joyce C. A. On the efficacy of isolating shoulder and elbow movements with a soft, portable, and wearable robotic device.

[64] Peng G., Fan X., Liu X. (2019). Design and control of a soft and wearable robotic glove for hand rehabilitation. *Journal of Medical Biomechanics*.

[65] Radder B., Prange-Lasonder, Gerdienke B. (2020). The effect of a wearable soft-robotic glove on motor function and functional performance of older adults. *Assistive Technology*.

[66] Fajardo J., Neto A. R., Silva W., Gomes M., Fujiwara E., Rohmer E. A wearable robotic glove based on optical FMG driven controller.

[67] Setiawan J. D., Ariyanto M., Munadi M., Muhammad M., Adam G., Wahyu C. (2020). Grasp posture control of wearable extra robotic fingers with flex sensors based on neural network. *Electronics*.

[68] Rahman M. H., Rahman M. J., Cristobal O. L., Saad M., Kenne J. P., Archambault P. S. (2015). Development of a whole arm wearable robotic exoskeleton for rehabilitation and to assist upper limb movements. *Robotica*.

[69] Cho Y. K., Kim K., Ma S., Ueda J. A robotic wearable exoskeleton for construction worker’s safety and health.

[70] Sandison M., Phan K., Casas R. HandMATE: wearable robotic hand exoskeleton and integrated android app for at home stroke rehabilitation.

[71] Hunte K., Chen S., Yi J. Assist-as-needed control of a wearable lightweight knee robotic device.

[72] Lopes J., Pinheiro C., Figueiredo J., Reis L. P., Santos C. P. Assist-as-needed impedance control strategy for a wearable ankle robotic orthosis. *‏*.

[73] Papapicco V., Parri A., Martini E., Bevilacqua V., Crea S., Vitiello N. Locomotion mode classification based on support vector machines and hip joint angles: a feasibility study for applications in wearable robotics.

